# Clinical implication of disturbed left atrial phasic functions in the heterogeneous population associated with hypertension or atrial fibrillation

**DOI:** 10.1186/s12947-019-0175-x

**Published:** 2019-11-12

**Authors:** Mengruo Zhu, Haiyan Chen, Yang Liu, Xianhong Shu

**Affiliations:** 1 Department of Echocardiography, Zhongshan Hospital, Fudan University, Shanghai, 200032 China; 2 Department of Cardiology, Zhongshan Hospital, Fudan University, Shanghai, 200032 China; 3 Shanghai Institute of Cardiovascular Diseases, Shanghai, 200032 China; 4 Shanghai Institute of Medical Imaging, Shanghai, 200032 China

**Keywords:** Atrial fibrillation, Hypertension, Left atrial, Phasic function, Speckle tracking echocardiography

## Abstract

**Background:**

To evaluate left atrial (LA) phasic functions in patients with hypertension and/or paroxysmal atrial fibrillation (PAF) and its clinical significance.

**Methods:**

LA strain was studied in 77 patients (25 hypertension, 24 lone AF, and 28 with both hypertension and PAF) and 28 controls using two-dimensional speckle-tracking echocardiography (2D STE). The following indexes during atrial reservoir, conduit and pump phase were analyzed respectively: (1) peak atrial longitudinal strain (PALS) and strain rate (PALSR), (2) the standard deviation of time to PALS and PALSR of all LA segments (TpS-SD% and TpSR-SD%).

**Results:**

Compared with controls, PALS_res_, PALS_cond_ and PALSR_cond_ were significantly reduced in patients with isolated hypertension (all *P* < 0.01) but no significant differences were observed in PALS_pump_, PALSR_pump_ and TpS_pump_-SD% between them (all *P* > 0.05). PALS_pump_, PALSR_pump_ and PALSR_res_ were significantly lower in patients with both hypertension and PAF than in those with isolated hypertension (all *P* < 0.05). PALS and PALSR were significantly decreased, and TpS-SD% was significantly increased during each phase in lone AF patients than in controls (all *P* < 0.05), and PALSR_pump_ was further depressed in patients with both hypertension and PAF (*P* = 0.029). PALSR_cond_ ≤ 1.475 s^− 1^ combined with TpS_pump_-SD% ≥ 3.25% (sensitivity, 85%; specificity, 71%; AUC = 0.845, *P* < 0.001) could distinguish lone AF from healthy subjects effectively, while in hypertensive patients, PALS_pump_ ≤ 14.2% was found to be an independent differentiator for occurrence of AF or not with sensitivity of 81% and specificity of 84% (AUC = 0.838, *P* < 0.001). LAVI≥29.3 mL/m^2^ was an independent characteristic for reflecting different LA remodeling in lone AF or hypertension with AF.

**Conclusions:**

The impairment of LA phasic functions was varied in patients with hypertension and/or AF. The disturbed LA phasic functions were proved to have independent abilities of differential diagnosis in this heterogeneous population associated with hypertension or AF.

## Introduction

Hypertension and atrial fibrillation (AF) are both associated with left atrial (LA) structural and functional abnormalities. LA enlargement in the patients with hypertension or AF is a common clinical phenomenon. However, the prognostic importance of LA phasic functions has more recently been recognized [1]. The LA modulates left ventricular (LV) filling through three components: reservoir, conduit and booster pump phase. Previous work has demonstrated the relationship between hypertension and LA dysfunction [2], and AF episodes always result in loss of LA pump function, but what are the differences of the impact on LA phasic functions between the coexistence of both conditions and isolated hypertension or AF was not well elucidated. Speckle tracking echocardiography (STE) is a feasible technique for the assessment of LA phasic functions by quantifying myocardial deformation performance and segmental myocardial motion synchrony [3]. Strain and strain rate (SR) reflect different aspects of myocardial deformation. The SR is the rate by which the deformation occurs (i.e. deformation per time unit). The strain, which equals the time integral of the SR, is deformation of an object relative to its original length. Early detection of phasic LA dysfunction can be indicated from the decreased strain, SR and advanced LA dyssynchrony.

In this study, we aimed to, firstly, explore different changes in LA phasic functions of patients with hypertension, paroxysmal AF (PAF), or both, and secondly, to evaluate the clinical significance of disturbed LA phasic functions in the heterogeneous population associated with hypertension or AF.

## Methods

### Study Population

Patients affected by hypertension and/or PAF, consecutively referred to our hospital from May 2018 to December 2018, were prospectively recruited for this study. Hypertension was diagnosed as systolic blood pressure ≥ 140 mmHg and/or diastolic blood pressure ≥ 90 mmHg on three or more occasions of different day, or as the current use of antihypertensive drugs in the presence of a documented history of hypertension according to the guidelines [4]. PAF was defined as AF, episodes confirmed by at least one electrocardiography (ECG) within a year, that terminates spontaneously or with intervention within 7 days of onset according to the guidelines [5]. Lone AF [5–9] referred to AF develops in a subset of younger persons (age ≤ 60 years) without clinical or echocardiographic evidence of cardiopulmonary disease (including hypertension, coronary heart disease) or any known risk factors for AF (including diabetes mellitus, obesity, increased alcohol consumption, sleep apnea et al). Only the ones with hypertension as a possible causative factor for AF and the lone AF individuals were included in the study. Patients with other known causes of AF such as coronary heart disease, heart failure, prior heart surgery, structural heart disease, thyroid dysfunction or renal failure were excluded from the study. Other exclusion criteria were LV ejection fraction (EF) < 50%, non-sinus rhythm during examination, moderate or severe valvular disease, hypertrophic cardiomyopathy, any other arrhythmia, history of ablation procedure, pacemaker implantation or cardiopulmonary surgery, chronic obstructive pulmonary disease and inadequate echocardiographic images. A total of 77 patients met the selection criteria during the period of enrollment. Within the study population, 25 patients had isolated hypertension but not PAF, 24 had lone AF, and 28 hypertensive patients with new-onset PAF, namely, those patients who have been first documented PAF episodes after known a history of hypertension. The control group consisted of 28 healthy individuals who came to our hospital for health check-up, without history of hypertension, PAF or other cardiovascular or systemic disease and with normal findings on clinical examination, ECG, and echocardiography.

The study protocol was approved by the local ethics committee, and informed consent was obtained from all patients before participation.

### Echocardiographic acquisition

All subjects, with a synchronous ECG connected, underwent a complete and standard transthoracic echocardiography (TTE) using a Philips iE33 ultrasound machine equipped with a S5-1 probe (Philips Medical Systems, Eindhoven, The Netherlands). All measurements were given as the average values of 3 consecutive cardiac cycles.

All measurements and evaluation were performed according to the guidelines of American society of echocardiography [10]. The LVEF was measured using the modified Simpson’s biplane method. Transmitral peak early diastolic filling velocity E was recorded by pulsed-wave Doppler at the tips of the mitral valve leaflets in an apical four-chamber view. Tissue Doppler imaging was applied in the pulsed-wave Doppler mode to record the mitral annulus peak early diastolic velocities (e’) at the septal and lateral positions. E/e’ was calculated as E divided by the average of the septal and lateral e’ velocities. LA volumes were calculated from the apical four- and two-chamber views of the LA using the biplane method of the discs. The maximum LA volume was indexed by dividing the body surface area to acquire the LA volume index (LAVI).

### Two-dimensional Speckle Tracking Analyses

Two-dimensional (2D) apical four-chamber, two-chamber and long-axis views acquired with at least 60 frames per second were digitized during five consecutive cardiac cycles in cine-loop format.

**Fig. 1:**
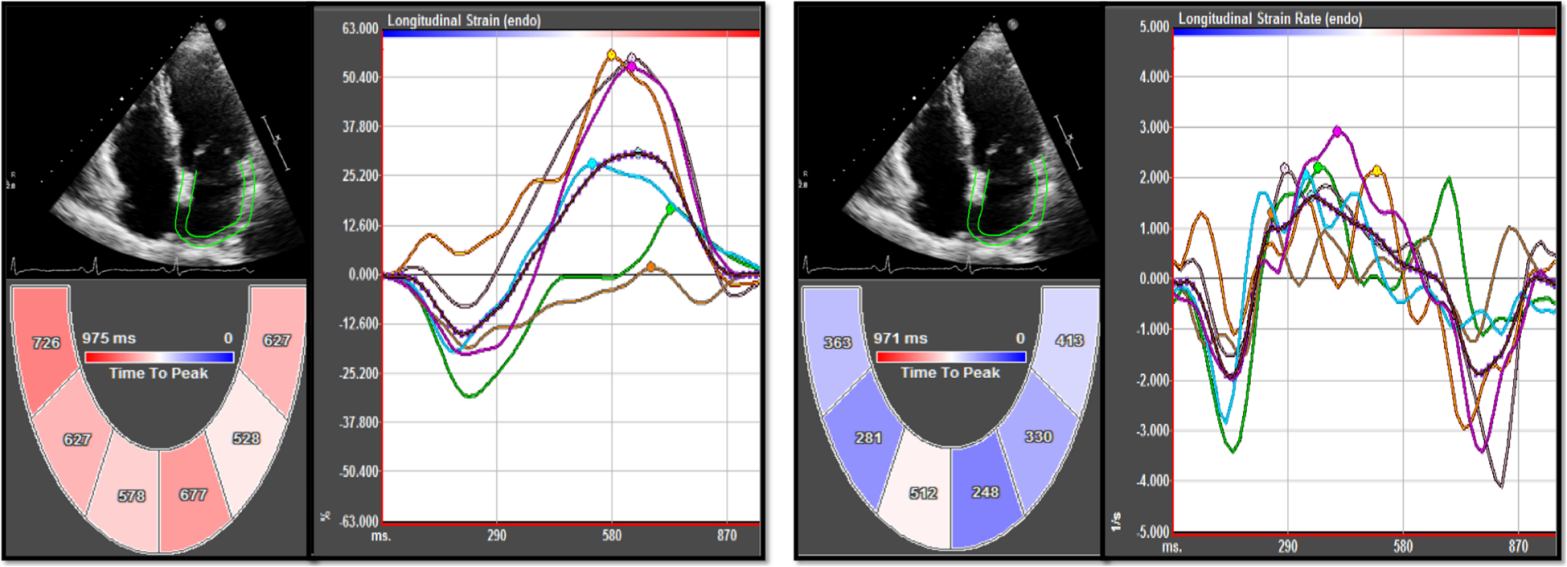
Pattern of left atrial strain (left) and strain rate (right) curves obtained from the apical four-chamber view in an example subject of our study population

**Fig. 2:**
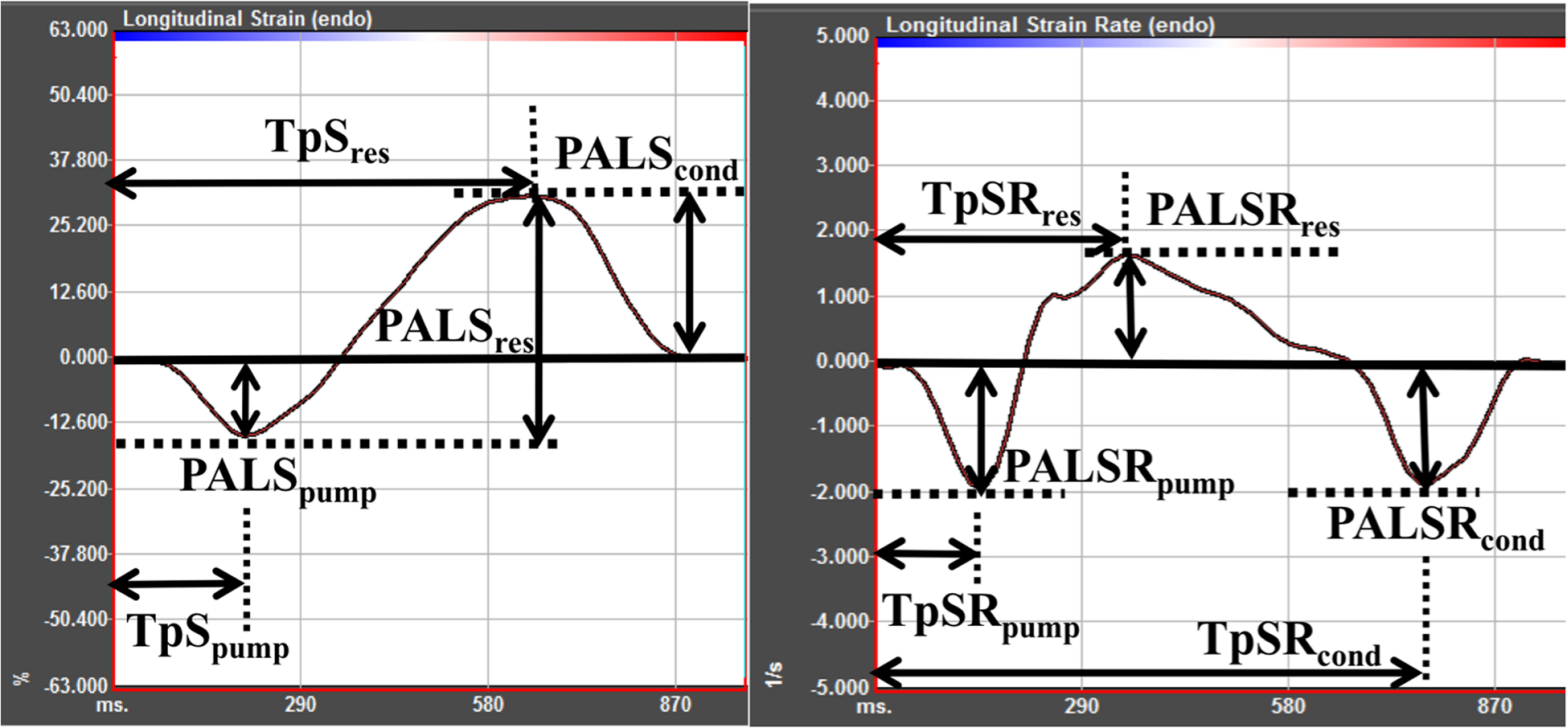
Schematic diagram of measurements of peak atrial longitudinal strain, strain rate (PALS, PALSR) and time to PALS, PALSR (TpS, TpSR) from left atrial strain and strain rate curves. Cond, atrial conduit phase; res, atrial reservoir phase; pump, atrial booster pump phase

Views were imported to the 2D speckle-tracking workstation, TomTec-Image Arena 4.0 (2D Cardiac Performance Analysis; TomTec Imaging System, Munich, Germany). Each view was analyzed according to the following steps: using P wave onset of the ECG as the reference point and selecting 3 cardiac cycles. Next, tracing the LA endocardial surface manually by a point-and-click approach. An epicardial surface tracing was automatically generated by the system, creating a region of interest which could be manually adjusted to cover the full thickness of the LA wall. Before processing, a cine loop preview feature visually confirmed that the internal line followed the LA endocardium throughout the 3 cardiac cycles. The software divided the LA wall into 6 segments in each view, and automatically generated longitudinal strain and SR curves of 6 segments and the average curve of 6 segments (Fig. 1). The following indexes were measured from LA strain and SR curves (Fig. 2): (1) peak atrial longitudinal strain and SR during atrial booster pump phase (PALS_pump_, PALSR_pump_), reservoir phase (PALS_res_, PALSR_res_), and conduit phase (PALS_cond_, PALSR_cond_) from average strain and SR curves of 6 segments in the apical four-chamber and two-chamber views, respectively; (2) time to PALS (TpS) and time to PALSR (TpSR), with the referent point at the onset of P-wave, of LA 15 segments in apical four-chamber, two-chamber and long-axis (including 3 segments of LA posterior wall and excluding 3 segments of aortic wall) views during each phase (TpS_pump_, TpS_res_, TpSR_pump_, TpSR_res_ and TpSR_cond_). The LA dyssynchrony index in each phase was defined as the standard deviation of 15 TpS or 15 TpSR parameters in this phase which was corrected by the RR interval duration (TpS_pump_-SD%, TpS_res_-SD%, TpSR_pump_-SD%, TpSR_res_-SD% and TpSR_cond_-SD%). All PALS and PALSR indexes were obtained by averaging values measured in the apical four-chamber and two-chamber views. Calculated the proportion of reservoir deformation (PALS_res_) contributed to conduit phase (PALS_cond_/PALS_res_ ratio) and pump phase (PALS_pump_/PALS_res_ ratio), as well as the ratio of the pump phasic component and the conduit phasic component (PALS_pump_/PALS_cond_ ratio and PALSR_pump_/PALSR_cond_ ratio). The ratio indices were used as indexed parameters of conduit and pump function for further assessment of phasic functions. Both three strain ratios (pump/res, pump/cond, cond/res) and one SR ratio (pump/cond) were calculated for comparing two components of LA emptying function (early passive and later active) among the four study groups.

### Inter- and intra-observer variability

**Fig. 3:**
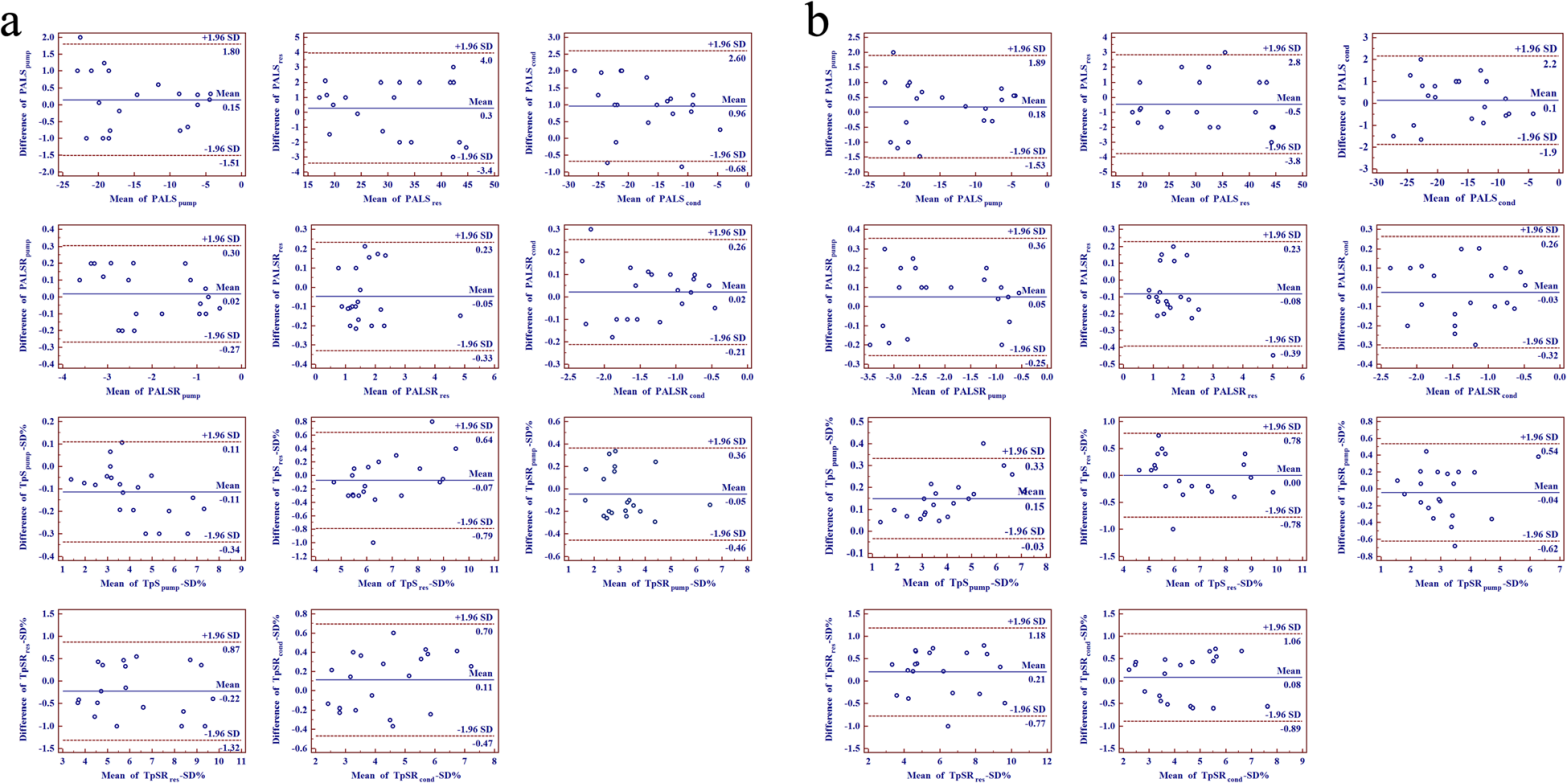
Bland-Altman analysis for inter-observer variability (**a**) and inter-observer variability (**b**) of left atrial strain and strain ratio indexes measurements. Solid line represents bias and dotted lines represent 95% limits of agreement for measurements performed in 20 patients. The bias was assessed by the mean of 20 differences of two measurements. The 95% confidence interval was calculated as ±1.96 SDs from the mean. Overall, small differences were observed for all left atrial strain and strain ratio indexes measurements because most of the differences were within the range of 95% limits of agreement, which suggesting good repeatability and reproducibility in LA strain and strain ratio indexes. Cond, atrial conduit phase; PALS, peak atrial longitudinal strain; PALSR, peak atrial longitudinal strain rate; pump, atrial booster pump phase; res, atrial reservoir phase; TpS-SD%, the standard deviation of time to peak atrial longitudinal strain corrected by RR interval; TpSR-SD%, the standard deviation of time to peak atrial longitudinal strain rate corrected by RR interval

Inter- and intra-observer variability of LA strain and strain ratio indexes measurements were assessed using Bland-Altman plots (Fig. 3) with data from 20 patients, 5 patients randomly selected from each group respectively, examined twice by one observer twice who was blinded to the results of the previous measurements and by a second observer who was blinded to the values obtained by the first observer, respectively. Overall, small differences were observed for all left atrial strain and strain ratio indexes measurements because most of the differences were within the range of 95% limits of agreement, which suggesting good repeatability and reproducibility in LA strain and strain ratio indexes.

### Statistical analysis

Continuous data were presented as the mean ± SD and dichotomous data as a percentage. Between-group comparisons of continuous variables were performed using one-way analysis of variance (ANOVA), followed by the Bonferroni post hoc test to adjust for multiple comparisons, when normality and homogeneity of variance assumptions are satisfied; otherwise the equivalent non-parametric tests were used when Kolmogorov-Smirnov was in favor of absence of normal distribution or the Levene’s test was in favor of absence of homogeneity of variance. Categorical variables were compared using chi-square tests or Fisher’s exact tests as appropriate. Logistic regression analysis was performed to identify independent differentiators in the heterogeneous population. Significant variables selected in univariate analysis were entered into the multivariate logistic regression analysis. Receiver operating characteristic (ROC) curve was constructed to determine the optimal cut-off value which combine the higher value of specificity plus sensitivity.

A two-sided *P* value < 0.05 was accepted as indicating statistical significance. All data were analyzed using SPSS version 22.0 (SPSS Inc., Chicago, IL, USA) and MedCalc version 12.5.0.0 (MedCalc Software, Mariakerke, Belgium).

## Results

### General Characteristics

Main clinical and conventional echocardiographic characteristics of the four study groups were shown in Table 1**.** No significant difference in age, sex and heart rate (HR) was observed among the four study groups. Indexed LV mass (LVMI), LAVI and E/e’ ratio were increased in hypertensive patients than normotensive patients (all *P* < 0.05), but no significant differences was found between hypertensive patients with PAF and those without PAF. Hypertensive patients with PAF had longer period of known hypertension as compared with those without PAF.

**Table 1:** Main clinical and conventional echocardiographic characteristics of the four study groups

Variable	Controls (*n* = 28)	Isolated HTN group (*n* = 25)	Lone AF group (*n* = 24)	HTN and PAF group (*n* = 28)	*P*
Age, years	56.7 ± 9.3	58.3 ± 8.9	53.8 ± 6.7	60.9 ± 6.6	0.386
Female gender, n (%)	13 (46%)	14 (56%)	13 (54%)	8 (29%)	0.166
Height, cm	166.8 ± 8.1	163.8 ± 6.7	170.0 ± 10.1	165.8 ± 7.4	0.061
Weight, kg	62.6 ± 9.6	62.2 ± 10.7	65.8 ± 10.0	65.7 ± 9.4	0.403
Body mass index, kg/m2	22.29 ± 2.53	23.06 ± 2.79	22.26 ± 2.36	23.83 ± 2.42	0.079
Body surface area, m2	1.70 ± 0.16	1.67 ± 0.17	1.77 ± 0.17	1.73 ± 0.15	0.180
Heart rate, beats/min	73.0 ± 7.7	70.5 ± 7.3	67.4 ± 11.5	68.5 ± 6.5	0.108
SBP, mm Hg	114.1 ± 6.9	159.0 ± 13.5*	113.9 ± 6.9	162.1 ± 8.8*	0.000
DBP, mm Hg	83.2 ± 3.7	100.4 ± 5.6*	80.6 ± 4.7	99.6 ± 7.3*	0.000
History of HTN, years	–	8.0 ± 4.6	–	11.9 ± 4.5#	0.003
History of AF, years	–	–	4.5 ± 3.9	5.4 ± 4.5	0.468
Current smoker, n (%)	5 (18%)	10 (36%)	–	9 (32%)	0.198
Current drinker, n (%)	4 (7%)	5 (28%)	–	8 (46%)	0.418
Medications, n (%)					
ACEI or ARB	–	13 (52%)	–	16 (57%)	0.707
β-blockers	–	7 (28%)	–	6 (21%)	0.579
Calcium antagonists	–	6 (24%)	–	6 (21%)	0.823
Antiplatelet	–	–	8 (33%)	11 (39%)	0.657
Anticoagulant agent	–	–	11 (46%)	15 (54%)	0.578
Antiarrhythmic drug	–	–	12 (50%)	17 (61%)	0.438
Diuretics	–	5 (20%)	3 (13%)	8 (46%)	0.360
Statins	–	8 (32%)	4 (17%)	11 (39%)	0198
Mild mitral regurgitation, n (%)	–	4 (16%)	7 (29%)	10 (36%)	0.266
Mild aortic regurgitation, n (%)	–	8 (32%)	3 (13%)	11 (39%)	0.093
LVEF, %	64.16 ± 2.79	63.65 ± 4.12	63.54 ± 4.13	63.86 ± 3.29	0.936
LVMI, g/m2	81.44 ± 7.26	108.52 ± 12.26*	77.41 ± 6.87	109.60 ± 19.21*	0.000
E/e’ ratio	8.24 ± 1.88	10.51 ± 2.85*	9.74 ± 2.76	10.96 ± 8.05*	0.007
LAVI, mL/m2	25.73 ± 5.66	31.93 ± 8.62*	27.60 ± 8.47	33.16 ± 10.08*	0.007

*ACE* Angiotensin converting enzyme, *AF* Atrial fibrillation, *ARB* Angiotensin receptor blocker, *DBP* Diastolic blood pressure, *HTN* Hypertension, *LAVI* Left atrial volume index, *LVEF* Left ventricular ejection fraction, *LVMI* Left ventricular mass index, *PAF* Paroxysmal atrial fibrillation, *SBP* Systolic blood pressure

*P < 0.05 vs. controls, #*P* < 0.05 vs. isolated HTN group

Data are expressed as mean ± SD or as number (percentage)

### LA Phasic Mechanical Functions

**Fig. 4:**
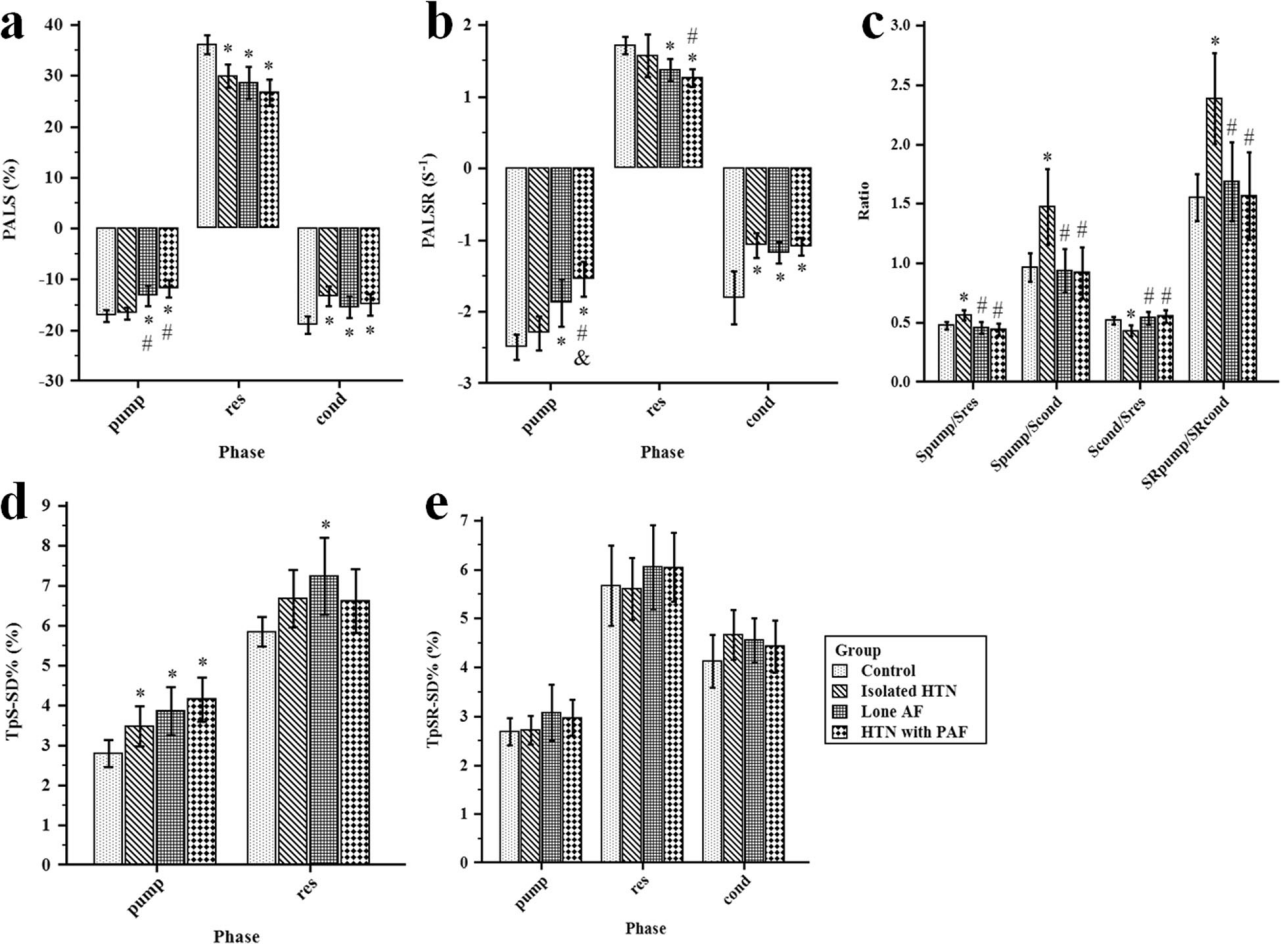
Comparisons of left atrial mechanical functional indexes in the four study groups. **a** Comparisons of PALS in each phase among the four study groups; **b** Comparisons of PALSR in each phase among the four study groups; **c** Comparisons of LA strain ratio and strain rate ratio indexes among the four study groups; **d** Comparisons of TpS-SD% in atrial pump and reservoir phase among the four study groups; **e** Comparisons of TpSR-SD% in each phase among the four study groups. AF, atrial fibrillation; cond, atrial conduit phase; HTN, hypertension; PAF, paroxysmal atrial fibrillation; PALS, peak atrial longitudinal strain; PALSR, peak atrial longitudinal strain rate; pump, atrial booster pump phase; res, atrial reservoir phase; TpS-SD%, the standard deviation of time to peak atrial longitudinal strain corrected by RR interval; TpSR-SD%, the standard deviation of time to peak atrial longitudinal strain rate corrected by RR interval. *P < 0.05 vs. controls; #*P* < 0.05 vs. isolated HTN group; &*P* < 0.05 vs. lone AF group

The comparisons of phasic LA mechanical functional parameters among the four study groups were shown in Tables 2 and Fig. 4**.**

**Table 2:** LA phasic mechanical functional indexes in the four study groups

Variable	Controls (*n* = 28)	Isolated HTN group (*n* = 25)	Lone AF group (*n* = 24)	HTN with PAF group (*n* = 28)	*P*
LA strain indexes (%)					
PALS_pump_	−17.13 ± 2.94	−16.65 ± 2.90	−13.73 ± 5.06*#	−11.62 ± 4.19*#	0.000
PALS_res_	36.10 ± 4.90	29.95 ± 5.56*	29.45 ± 7.53*	26.49 ± 6.64*	0.000
PALS_cond_	−18.96 ± 4.56	−13.30 ± 4.81*	− 15.73 ± 5.26*	−14.87 ± 5.30*	0.001
LA strain rate indexes (s^−1^)					
PALSR_pump_	−2.49 ± 0.45	−2.30 ± 0.57	−1.88 ± 0.78*	−1.54 ± 0.62*#&	0.000
PALSR_res_	1.71 ± 0.30	1.57 ± 0.71	1.43 ± 0.34*	1.23 ± 0.31*#	0.001
PALSR_cond_	−1.80 ± 0.95	−1.07 ± 0.41*	− 1.18 ± 0.35*	−1.10 ± 0.33*	0.000
LA strain ratio and strain rate ratio indexes					
PALS_pump_/PALS_res_	0.48 ± 0.08	0.57 ± 0.11*	0.46 ± 0.13#	0.44 ± 0.13#	0.001
PALS_cond_/PALS_res_	0.52 ± 0.08	0.43 ± 0.11*	0.54 ± 0.13#	0.56 ± 0.13#	0.001
PALS_pump_/PALS_cond_	0.96 ± 0.31	1.48 ± 0.77*	0.97 ± 0.47#	0.90 ± 0.53#	0.001
PALSR_pump_/PALSR_cond_	1.55 ± 0.50	2.39 ± 0.93*	1.79 ± 0.81#	1.52 ± 0.90#	0.000
LA synchrony indexes (%)					
TpS_pump_-SD%	2.80 ± 0.89	3.48 ± 1.21	3.75 ± 1.33*	4.19 ± 1.47*	0.001
TpS_res_-SD%	5.84 ± 0.95	6.68 ± 1.76	7.22 ± 2.39*	6.70 ± 2.03	0.071
TpSR_pump_-SD%	2.69 ± 0.72	2.72 ± 0.72	2.96 ± 1.19	3.06 ± 1.12	0.386
TpSR_res_-SD%	5.67 ± 2.12	5.61 ± 1.54	5.67 ± 1.82	6.29 ± 1.93	0.463
TpSR_cond_-SD%	4.13 ± 1.40	4.67 ± 1.24	4.47 ± 1.09	4.49 ± 1.33	0.480

*AF* Atrial fibrillation, *cond* Atrial conduit phase, *HTN* Hypertension, *LA* Left atrial, *PAF* Paroxysmal atrial fibrillation, *PALS* Peak atrial longitudinal strain, *PALSR* Peak atrial longitudinal strain rate, *pump* Atrial pump phase, *res* Atrial reservoir phase, *TpS-SD%* The standard deviation of time to peak atrial longitudinal strain corrected by RR interval, *TpSR-SD%* The standard deviation of time to peak atrial longitudinal strain rate corrected by RR interval

*P < 0.05 vs. controls, #*P* < 0.05 vs. isolated HTN group, &*P* < 0.05 vs. lone AF group

Data are expressed as mean ± SD

#### LA Strain and SR

PALS_res_, PALS_cond_ and PALSR_cond_ were significantly lower in patients with isolated hypertension than in controls (*P* = 0.003, *P* < 0.001 and *P* < 0.001, respectively), whereas no significant differences were observed in PALS_pump_ and PALSR_pump_ between them (both *P* > 0.05) (Fig. 4a, b). Besides, when compared with other three groups, the patients with isolated hypertension showed significantly higher PALS_pump_/PALS_res_ ratio (vs. controls: *P* = 0.034, vs. lone AF patients: *P* = 0.006, vs. patients with both hypertension and PAF: *P* = 0.001), PALS_pump_/PALS_cond_ ratio (vs. controls: *P* = 0.005, vs. lone AF patients: *P* = 0.005, vs. patients with both hypertension and PAF: *P* = 0.002) and PALSR_pump_/PALSR_cond_ ratio (vs. controls: *P* = 0.002, vs. lone AF patients: *P* = 0.018, vs. patients with both hypertension and PAF: *P* = 0.002), and significantly lower PALS_cond_/PALS_res_ (vs. controls: *P* = 0.034, vs. lone AF patients: *P* = 0.006, vs. patients with both hypertension and PAF: *P* = 0.001) (Fig. 4c). Similar to that findings in patients with isolated hypertension, PALS_res_, PALS_cond_ and PALSR_cond_ were also significantly lower in patients with both hypertension and PAF than in controls (*P* < 0.001, *P* = 0.021 and *P* < 0.001, respectively). In addition, PALS_pump_, PALSR_pump_ and PALSR_res_ were also significantly reduced in patients with both hypertension and PAF when compared with controls (all *P* < 0.001) and those with isolated hypertension (*P* < 0.001, *P* < 0.001 and *P* = 0.034, respectively) (Fig. 4a, b).

PALS and PALSR during each phase were significantly reduced in lone AF patients when compared with controls (all *P* < 0.05) (Fig. 4a, b), and PALSR_pump_ was further reduced in patients with both hypertension and PAF in comparison with that in lone AF patients (*P* = 0.029) (Fig. 4b). PALS_pump_ was also significantly lower in lone AF patients when compared with patients with isolated hypertension (*P* = 0.014) (Fig. 4a).

#### LA Dyssynchrony

Both TpS_pump_-SD% and TpS_res_-SD% were significantly higher in patients with lone AF than in controls (*P* = 0.017 and *P* = 0.041, respectively), and only TpS_pump_-SD% was higher in patients with both hypertension and PAF than in controls (*P* = 0.001) (Fig. 4d). No significant differences were observed in TpS_pump_-SD% and TpS_res_-SD% between patients with isolated hypertension and controls (Fig. 4d). No significant differences in TpSR-SD% during each phase were observed among the four study groups (Fig. 4e).

### Analyses to identify differentiators in the heterogeneous population

**Fig. 5:**
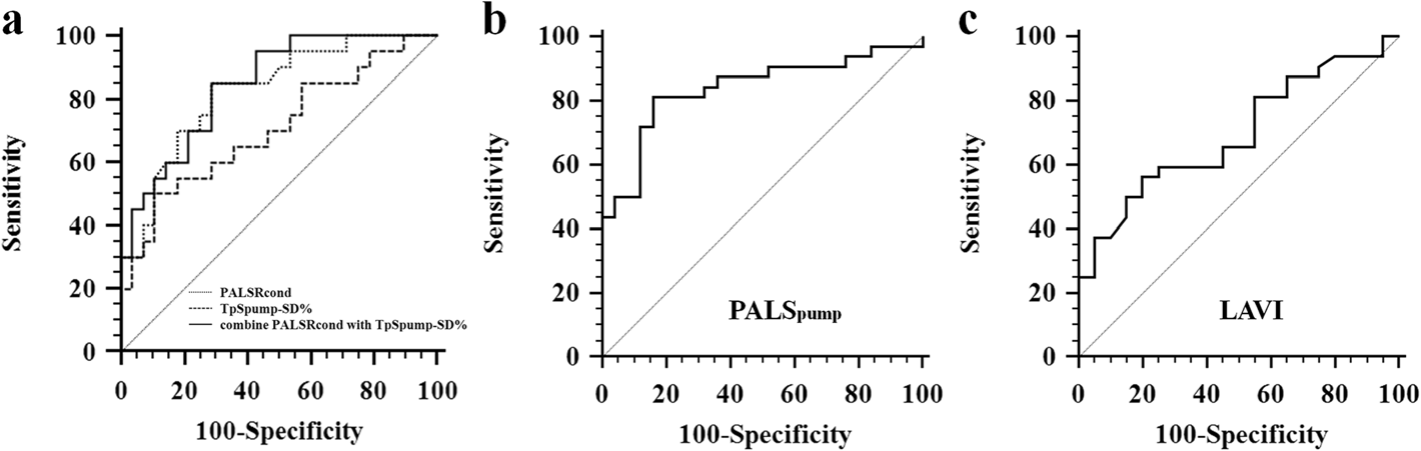
Receiver operating characteristic (ROC) curves of differentiators for differential diagnosis in the heterogeneous population. **a** ROC analysis to differentiate lone AF from healthy controls; **b** ROC analysis to differentiate isolated HTN group and HTN with PAF group; **c** ROC analysis to differentiate between lone AF group and HTN with PAF group. AF, atrial fibrillation; cond, atrial conduit phase; HTN, hypertension; LAVI, left atrial volume index; PAF, paroxysmal atrial fibrillation; PALS, peak atrial longitudinal strain; PALSR, peak atrial longitudinal strain rate; pump, atrial booster pump phase; TpS-SD%, the standard deviation of time to peak atrial longitudinal strain corrected by RR interval

Logistic regression analyses were performed to differentiate lone AF from healthy subjects (Table 3) and to identify differentiators for occurrence of AF in hypertensive patients (Table 4). All variables significantly associated with occurrence of AF in univariate analysis were involved in multivariate analysis. PALSR_cond_ (odds ratio (OR) 0.006, 95% confidence interval (CI) 0.000-0.581, *P* = 0.028) and TpS_pump_-SD% (OR 2.294, 95% CI 1.228-4.285, *P* = 0.009) were found to have independent ability to differentiate lone AF from healthy subjects in our study (Table 3). Fig. 5a shows the ROC curves constructed using the two differentiators and their combination for determining their abilities of differential diagnosis. The optimal cutoff value of PALSR_cond_ was recommended as 1.475 s^-1^ with sensitivity of 85% and specificity of 71% (area under the ROC curve (AUC) = 0.825, *P* < 0.001), and the optimal cutoff value of TpS_pump_-SD% was recommended as 3.25% with sensitivity of 60% and specificity of 71% (AUC = 0.707, *P* = 0.015). The combination of the two differentiators, PALSR_cond_ ≤ 1.475 s^-1^ and TpS_pump_-SD% ≥ 3.25%, yielded sensitivity of 85%, specificity of 71% and increased AUC = 0.845 (*P* < 0.001) than individual parameters. While in hypertensive patients (Table 4), PALS_pump_ (OR 0.620, 95% CI 0.457-0.843, *P* = 0.002) was found to be an independent differentiator for occurrence of AF or not. ROC analysis identified the optimal cutoff value of PALS_pump_ as 14.2% with sensitivity of 81% and specificity of 84% (AUC = 0.838, *P* < 0.001) for distinguishing the hypertensive patients with AF from those without AF (Fig. 5b).

**Table 3:** Univariate and multivariate logistic regression analysis to differentiate lone AF from healthy controls

Variable	Univariate Analysis			Multivariate Analysis		
	OR	95% CI	*P*	OR	95% CI	*P*
Age	0.967	0.911–1.027	0.273			
Female gender	0.635	0.212–1.902	0.417			
Heart rate	0.941	0.884–1.001	0.056			
LVEF	0.980	0.837–1.147	0.800			
LVMI	0.921	0.847–1.001	0.053			
E/e’ ratio	1.386	1.048–1.832	0.022	1.372	0.960–1.960	0.083
LAVI	1.016	0.926–1.114	0.743			
PALS_pump_	0.801	0.672–0.954	0.013	0.701	0.418–1.173	0.176
PALS_res_	0.836	0.740–0.945	0.004	1.189	0.838–1.689	0.333
PALS_cond_	0.870	0.764–0.990	0.034	1.160	0.808–1.665	0.422
PALSR_pump_	0.267	0.090–0.786	0.017	2.718	0.160–46.264	0.489
PALSR_res_	0.052	0.005–0.505	0.011	2.957	0.032–276.719	0.640
PALSR_cond_	0.028	0.003–0.250	0.001	0.006	0.000–0.581	0.028
TpS_pump_-SD%	2.310	1.203–4.436	0.012	2.294	1.228–4.285	0.009
TpS_res_-SD%	1.653	1.082–2.524	0.020	1.345	0.927–1.954	0.119
TpSR_pump_-SD%	1.368	0.723–2.589	0.335			
TpSR_res_-SD%	1.000	0.745–1.340	0.997			
TpSR_cond_-SD%	1.241	0.784–1.964	0.357			

*AF* Atrial fibrillation, *CI* Confidence interval, *cond* Atrial conduit phase, *LAVI* Left atrial volume index, *LVEF* Left ventricular ejection fraction, *LVMI* Left ventricular mass index, *OR* Odds ratio, *PALS* Peak atrial longitudinal strain, *PALSR* Peak atrial longitudinal strain rate, *pump* Atrial pump phase, *res* Atrial reservoir phase, *TpS-SD%* The standard deviation of time to peak atrial longitudinal strain corrected by RR interval, *TpSR-SD%* The standard deviation of time to peak atrial longitudinal strain rate corrected by RR interval

**Table 4:** Univariate and multivariate logistic regression analysis to identify differentiators between isolated HTN group and HTN with PAF group

Variable	Univariate Analysis			Multivariate Analysis		
	OR	95% CI	*P*	OR	95% CI	*P*
Age	1.035	0.965–1.109	0.335			
Female gender	2.43	0.829–7.120	0.106			
Heart rate	0.968	0.891–1.051	0.436			
History of HTN	1.209	0.997–1.396	0.056			
SBP	1.026	0.976–1.079	0.311			
DBP	0.982	0.903–1.068	0.670			
LVEF	0.988	0.847–1.153	0.882			
LVMI	1.011	0.958–1.066	0.697			
E/e’ ratio	1.047	0.873–1.255	0.621			
LAVI	1.014	0.954–1.079	0.649			
PALS_pump_	0.702	0.583–0.846	0.000	0.620	0.457–0.843	0.002
PALS_res_	0.913	0.835–0.998	0.046	1.179	0.942–1.475	0.150
PALS_cond_	1.065	0.956–1.187	0.252			
PALSR_pump_	0.101	0.030–0.343	0.000	0.146	0.005–4.103	0.258
PALSR_res_	0.090	0.012–0.656	0.018	0.365	0.007–20.313	0.623
PALSR_cond_	1.221	0.282–5.282	0.790			
TpS_pump_-SD%	1.485	0.975–2.262	0.065			
TpS_res_-SD%	1.008	0.763–1.331	0.957			
TpSR_pump_-SD%	1.493	0.813–2.742	0.196			
TpSR_res_-SD%	1.252	0.916–1.711	0.159			
TpSR_cond_-SD%	0.897	0.594–1.356	0.607			

*CI* Confidence interval, *cond* Atrial conduit phase, *DBP* Diastolic blood pressure, *HTN* Hypertension, *LAVI* Left atrial volume index, *LVEF* Left ventricular ejection fraction, *LVMI* Left ventricular mass index, *OR* Odds ratio, *PAF* Paroxysmal atrial fibrillation, *PALS* Peak atrial longitudinal strain, *PALSR* Peak atrial longitudinal strain rate, *pump* Atrial pump phase, *res* Atrial reservoir phase, *TpS-SD%* The standard deviation of time to peak atrial longitudinal strain corrected by RR interval, *TpSR-SD%* The standard deviation of time to peak atrial longitudinal strain rate corrected by RR interval, *SBP* Systolic blood pressure

Logistic regression analysis was also performed to indicate the key differentiator between lone AF group and hypertension with AF group (Table 5). Multivariate analysis revealed LAVI (OR 1.077, 95% CI 1.021-1.136, *P* = 0.006) was an independent characteristic for reflecting different LA remodeling in two types of AF patients. The optimal cut-off value of LAVI was 29.3 mL/m^2^ (sensitivity, 60%; specificity, 75%; AUC = 0.695, *P* = 0.019) which determined by ROC curve (Fig. 5c).

**Table 5:** Univariate and multivariate logistic regression analysis to identify differentiators between lone AF group and HTN with PAF group

Variable	Univariate Analysis			Multivariate Analysis		
	OR	95% CI	P	OR	95% CI	*P*
Age	1.066	0.992–1.145	0.081			
Female gender	2.955	0.938–9.309	0.064			
Heart rate	1.014	0.953–1.077	0.665			
LVEF	1.024	0.881–1.190	0.755			
History of AF	1.052	0.919–1.205	0.462			
E/e’ ratio	1.135	0.944–1.363	0.177			
LAVI	1.088	1.015–1.166	0.017	1.077	1.021–1.136	0.006
PALS_pump_	0.901	0.792–1.025	0.114			
PALS_res_	0.940	0.865–1.021	0.143			
PALS_cond_	0.969	0.870–1.079	0.562			
PALSR_pump_	0.343	0.141–0.838	0.019	0.433	0.114–1.648	0.220
PALSR_res_	0.150	0.024–0.937	0.042	0.812	0.091–7.254	0.852
PALSR_cond_	0.474	0.085–2.644	0.394			
TpS_pump_-SD%	1.254	0.830–1.894	0.282			
TpS_res_-SD%	0.894	0.687–1.162	0.401			
TpSR_pump_-SD%	1.368	0.723–2.589	0.335			
TpSR_res_-SD%	1.000	0.745–1.340	0.997			
TpSR_cond_-SD%	1.241	0.784–1.964	0.357			

*AF* Atrial fibrillation, *CI* Confidence interval, *cond* Atrial conduit phase, *HTN* Hypertension, *LAVI* Left atrial volume index, *LVEF* Left ventricular ejection fraction, *OR* Odds ratio, *PAF* Paroxysmal atrial fibrillation, *PALS* Peak atrial longitudinal strain, *PALSR* Peak atrial longitudinal strain rate, *pump* Atrial pump phase, *res* Atrial reservoir phase, *TpS-SD%* The standard deviation of time to peak atrial longitudinal strain corrected by RR interval, *TpSR-SD%* The standard deviation of time to peak atrial longitudinal strain rate corrected by RR interval

## Discussion

STE can provide excellent visualization of the phasic LA mechanical function. In this study, we evaluated LA phasic functions using STE in patients with hypertension, PAF, or both and explored the differences of impact on phasic LA mechanical functions between patients with both hypertension and PAF, and those with isolated hypertension or lone AF, and then further to study the clinical implications of disturbed LA phasic functions in the heterogeneous population associated with hypertension or lone AF. Our findings suggest that (1) in early stage of hypertension, conduit function is the most severely impaired, followed by reservoir function, while booster pump function is still preserved and contribution proportion of pump phase shows a compensatory increase. (2) With the occurrence of AF causing decompensation in hypertensive patients, LA booster pump function is impaired and reservoir function is further depressed. (3) LA reservoir, conduit and booster pump function are impaired in lone AF patients even with normal LA size. The magnitude of this impairment in booster pump and reservoir phase is further increased in subjects with coexisting hypertension and PAF in comparison with those with isolated hypertension or lone AF. (4) Decreased conduit SR combined with advanced contractile dyssynchrony could further improve the accuracy of differentiating lone AF from healthy subjects, while in hypertensive patients with enlarged LA, decreased contractile strain was proved to have the independent capability of differential diagnosis for the occurrence of AF or not. As far as the differences of LA remodeling between two types of AF, LAVI was an independent characteristic for reflecting it.

### Disturbed LA Phasic Functions

LA function is known to be divided into three parts: reservoir, conduit, and booster pump function. The three components are mutual interdependence and can be redistributed to compensate for each other in order to maintain cardiac output in early stage of some pathophysiological conditions. In our study, the depression in PALS_res_, PALS_cond_ and PALSR_cond_ shown in hypertensive patients suggested that LA conduit function was the most severely impaired, followed by reservoir function. The relations of LV longitudinal strain and LA strain were described in previous studies [11–13] The impairment of LA reservoir and conduit strain were likely reflecting impaired LV longitudinal strain in hypertensive subjects. Hypertension caused an increase in LV wall stress, leading to myocyte hypertrophy and myocardial fibrosis, which resulted in impaired myocardial relaxation and increased LV diastolic stiffness, thereby further induce elevated LV diastolic filling pressure. The impairment of LA reservoir and conduit function better reflects the atrial response to increased ventricular filling pressures. Although LAVI was significantly larger in hypertensive patients than in controls, no significant differences were observed in PALS_pump_, PALSR_pump_ and TpS_pump_-SD% between them. This finding suggested that LA booster pump function was still preserved in early stage of hypertension in absence of AF. It is worthwhile to note that our results indicated that hypertensive patients showed a compensatory increasing contribution of booster pump phase to emptying function, whereas in normotensive individuals the LA emptying blood into LV occurred predominantly in atrial conduit phase. Although the absolute value of strain, SR and dyssynchrous in pump phase didn’t show significant differences between the patients with isolated hypertension and controls, an increased proportion of atrial active contractile strain was observed which compensated for the reduction in the proportion of conduit deformation to emptying function. As shown in our study, the indexed parameters of pump function, including PALS_pump_/PALS_res_ ratio, PALS_pump_/PALS_cond_ ratio and PALSR_pump_/PALSR_cond_ ratio, were significantly higher, while the indexed parameters of conduit function, namely, PALS_cond_ /PALS_res_ ratio, was significantly lower in patients with isolated hypertension than other three groups as a result of increased active contractility of atrial myocardium [14]. In the early filling of the LV, the LA acts as a conduit, passively emptying during LV relaxation, which is strongly influenced by LV compliance [15]. Hypertension is associated with changes in both LV compliance and relaxation, which influences the balance between early and late filling. After early filling and diastasis, the fall in passive volume shift from LA into LV, forces the atrium to both increase its stroke volume during contraction and recruit more contractile force to work against the increased ventricular pressure and to ensure efficient pumping [14, 16]. Augmented pump function is one of the mechanisms compensating for decreased early filling in patients with reduced LV compliance. An increase in LA active contractility is considered to be caused by the increase of LA volume——Frank-Starling’s law [17]. As a result of optimal use of the Frank-Starling mechanism of the atrial muscle, atrial shortening is augmented with chamber dilation until extreme dilation no longer provokes the Frank-Starling response [18].

Our study demonstrated that PALS_pump_ and PALSR_pump_ were lower in patients with both hypertension and PAF than in patients with isolated hypertension. Atrial myocardium being predisposed to increased load and wall stress for a long time, which are developing atrial myocardial fibrosis and dispersion in atrial electrical conductivity. The cumulative long-term effects of high blood pressure may be the underlying precipitating factor and the crucial point for AF. During AF, LA reservoir and conduit functions are impaired and the booster pump function is absent. In sinus rhythm, the atrial myocardial properties could hardly return to normal instantly. Our results also revealed the impairment on LA mechanical function in lone AF patients even with normal LA size, which manifested as not only reduced atrial myocardial deformation properties but also uncoordinated motion during each phase. This result was consistent with previous studies concerned with atrial impairment in AF [19]**.** Moreover, our findings extend prior work, confirming that PALSR_pump_ was further reduced in patients with both hypertension and PAF when compared to the lone AF patients. Extreme dilation and LA muscle fibrosis may account for the depressed LA booster pump function in hypertensive patients with PAF [20]. In addition, our results showed LA conduit function was not significantly different between the hypertensive patients with and without PAF, which are consistent with Barbier’ study [21]. However, Cui et al [22] suggested the occurrence of PAF in hypertensive patients is associated with enhanced LA reservoir and conduit function, while our results indicated that PALSR_res_ was reduced in hypertensive patients with PAF when compared to those hypertensive patients without PAF. Possible reasons for the contradictory results may be various patient clinical characteristics, for example, the hypertension duration, the clinical stages of AF, or the frequency and total number of PAF episodes.

### Clinical Implications in the Heterogeneous Population

Importance of LA phasic function evaluation is increasingly recognized for its incremental value in terms of prognosis and risk stratification in various disease states [1, 23–28]. We studied the clinical relevance and prognostic utility of disturbed LA phasic function in heterogeneous population associated with hypertension or AF. Differences in LA phasic function between different groups remained significant ability of differential diagnosis after adjustment for other confounders. These differences were also useful for the stratification of LA mechanical performance. Hypertension and AF are both important risk factors for stroke, heart failure and overall mortality [29–31]. LA enlargement and dysfunction induced by hypertension was found as independent determinants of new-onset AF [32, 33]. Because of high prevalence of hypertension, it appears to be the most common risk factor for AF and be responsible for more AF than any other risk factor [34, 35]. AF was associated with increased cardiovascular events in hypertensive patients, while hypertension increased the risk of stroke and cardiovascular mortality in patients with AF [36, 37]. Given hypertension is a modifiable risk factor for AF [38], we attempted to determine the threshold of phasic LA strain that distinguishes between isolated hypertension and hypertension with AF, which would be helpful to guide and monitor the progress of treatment of hypertension for preventing AF or reducing the risk of AF. In the current study, we demonstrated that in hypertensive patients, reduced PALS_pump_ was an independent differentiator for complications of AF or not after multivariate analysis. This result implied that LA mechanical deformation during pump phase was associated with higher risk for developing AF and more important than other factors in the development of AF. Therefore, we proposed atrial strain measurements as a stratification method in hypertension patients to select those at risk of imminent AF development because occurrence of AF was associated with impairment of LA myocardial properties. Remodeling of LA mechanical function in lone AF patients can be detected by 2D STE before structural remodeling. Decreased PALSR_cond_ combined with increased TpS_pump_-SD% can distinguish lone AF patients with normal LA size from healthy subjects, which suggesting cardiologist to guide patients who referred to hospital for episodic palpitation to have a 2D STE evaluation of phasic LA mechanical function. On the other hand, LAVI was proved to be the independent characteristic for reflecting different LA remodeling in two types of AF, which suggesting the occurrence and developing mechanism of AF associated with hypertension, i.e., irreversible LA enlargement with a histological change causing electrical changes in the atrium and then resulting in AF with LA dyssynchrony even during sinus rhythm.

### Limitations

There were several limitations of the present study. First, as dedicated software for LA strain analysis has not yet been released, we used the software for LV analysis to study LA strain. Second, the control group enrolled in our present study consisted of those healthy individuals who came to our hospital for health check-up, without history of cardiovascular or systemic disease and with normal findings on clinical examination, conventional ECG and echocardiography. However, we didn’t make more aggressive efforts, such as 24-hour Holter ECG, to identify if these healthy controls were asymptomatic PAF.

## Conclusions

We described patterns of phasic LA dysfunction in patients with hypertension or AF using 2D STE and demonstrated the differences in the disturbed LA phasic functions had significant ability of differential diagnosis in the heterogeneous population associated with hypertension or AF. A comprehensive evaluation for LA structure and function is feasible with 2D STE, which could provide prognostic information in clinical practice for disease and therapeutic monitoring, as well as risk stratification of patients with hypertension or AF.

## Data Availability

All datasets used and analyzed during the current study are available from the corresponding author on reasonable request.

## Abbreviations

2D: two-dimensional
AF: Atrial fibrillation
AUC: Area under the curve
CI: Confidence interval
EF: Ejection fraction
LA: Left atrial
LAVI: LA volume index
LV: Left ventricular
LVMI: Indexed LV mass
PAF: Paroxysmal atrial fibrillation
PAF: Paroxysmal atrial fibrillation
PALS: Peak atrial longitudinal strain
PALSR: Peak atrial longitudinal strain rate
ROC: Receiver operating characteristic
SR: Strain rat
STE: Speckle tracking echocardiography
TpSR-SD%: the standard deviation of time to peak atrial longitudinal strain rate corrected by RR interval
TpS-SD%: the standard deviation of time to peak atrial longitudinal strain corrected by RR interval
TTE: transthoracic echocardiography
